# An Update on Oxidative Damage to Spermatozoa and Oocytes

**DOI:** 10.1155/2016/9540142

**Published:** 2016-01-28

**Authors:** Chinyerum S. Opuwari, Ralf R. Henkel

**Affiliations:** Department of Medical Biosciences, University of the Western Cape, Robert Sobukwe Road, Bellville 7530, South Africa

## Abstract

On the one hand, reactive oxygen species (ROS) are mandatory mediators for essential cellular functions including the function of germ cells (oocytes and spermatozoa) and thereby the fertilization process. However, the exposure of these cells to excessive levels of oxidative stress by too high levels of ROS or too low levels of antioxidative protection will render these cells dysfunctional thereby failing the fertilization process and causing couples to be infertile. Numerous causes are responsible for the delicate bodily redox system being out of balance and causing disease and infertility. Many of these causes are modifiable such as lifestyle factors like obesity, poor nutrition, heat stress, smoking, or alcohol abuse. Possible correctable measures include foremost lifestyle changes, but also supplementation with antioxidants to scavenge excessive ROS. However, this should only be done after careful examination of the patient and establishment of the individual bodily antioxidant needs. In addition, other corrective measures include sperm separation for assisted reproductive techniques. However, these techniques have to be carried out very carefully as they, if applied wrongly, bear risks of generating ROS damaging the germ cells and preventing fertilization.

## 1. Introduction

One of the downfalls of all assisted reproduction techniques (ARTs), particularly intracytoplasmic sperm injection (ICSI), is thought to be the fact that genetically and/or chromosomally damaged spermatozoa may fertilize an oocyte by bypassing all the physiological selection barriers in place in the female organism to prevent such event (for review see [1]). The likelihood of using DNA-damaged spermatozoa in ICSI is much higher since the DNA fragmentation rate is significantly higher in patients with poor semen quality [2, 3] and the DNA damage cannot be recognized while selecting spermatozoa for this process [4] and since there is no practical, nonconsumptive test available that can successfully exclude DNA-damaged spermatozoa from being selected by the embryologist for the injection procedure [5, 6]. Besides affecting fertilization and the outcome of pregnancy, sperm DNA damage has a negative impact on the health of the offspring as uncorrected sperm DNA damage following zygote formation has the potential to create mutations/epimutations in the offspring [7]. This has been shown for intrauterine insemination (IUI),* in vitro* fertilization (IVF), and ICSI, and increased incidences of chromosomal abnormalities, minor and major birth defects, or early childhood cancers, particularly in the male offspring, have been linked to these procedures [8–16]. The level of sperm DNA fragmentation may give a sense of guidance as to the appropriate method of ARTs to employ especially between IVF and ICSI [17, 18]. As a result, numerous authors have suggested an introduction of sperm DNA damage testing into the andrological laboratory workup as an independent tool as DNA fragmentation apart from normal sperm morphology appears to be a reliable and more robust parameter than conventional semen analysis due to its low biological variability and thus is a useful biomarker that should be implemented in any andrological diagnostic workup [19–21]. Furthermore, sperm DNA damage appears to be linked to the most important checkpoints of fertility such as reduced fertilization rates, lower embryo quality and pregnancy rates, higher miscarriage rates, malformations, and childhood diseases [22].

Evidence continues to suggest that ART does increase risk of higher order pregnancy (with its inherent pre- and perinatal risks), prematurity and low birth weight, congenital malformations, in particular of the male urogenital system, and imprinting disorders [23–26]. Paternal sperm DNA damage has negative effects on the integrity of early embryonic development as the percentage of good quality embryos as well as implantation rates was significantly reduced in patients exhibiting high DNA damage [27] and it appears that the second and third mitoses are the sensitive periods [28]. Ghaleno et al. (2014) showed that the intracellular levels of hydrogen peroxide (H_2_O_2_) and superoxide (O_2_^−∙^) correlate negatively with impaired sperm mitochondrial membrane potential leading to poor-quality pronuclear embryos [29]. However, only H_2_O_2_ interfered with pronuclear formation. Reassuringly, evidence points away from an increased overall cancer risk or differences in neurodevelopmental outcomes. However, many unknowns remain, including future fertility and cardiovascular risks and risk of cerebral palsy [30].

On the other hand, very recent data suggest that the observed increased frequency of birth defects, congenital malformations, and chromosomal abnormalities after assisted reproduction might be due to confounders [31, 32]. It appears that the increase in birth defects because of increased sex chromosome abnormalities is due to the assisted reproduction protocol* per se* rather than to the biological perturbations [33]. Yet in the absence of indubitable evidence of no increased risks it is mandatory to investigate the possible influence of the male genome on the health of the offspring with a suitable test.

Considering the inconsistent data situation, the existence of a variety of different assays that test for different aspects of sperm DNA fragmentation [34], and a lack of standardization and clinical evaluation, which makes it difficult for clinicians and scientists to decide which assay would be the best for clinical implementation [21], it is mandatory in the best interest of both prospective parents and the future offspring to implement a test to evaluate the extent of sperm DNA damage. Furthermore, there is still an urgent need for scientists to understand the principle on which the respective assays are based and which aspects of DNA damage these tests measure [4]. The various tests employed for DNA damage, the level at which sperm DNA damage may occur, assay principles, and the advantages and disadvantages thereof have been reviewed [4].

In this context, numerous reports have been published on the impact of oxidative stress on spermatozoa (for review see [35–38]) with its beneficial and detrimental effects of reactive oxygen species (ROS) like H_2_O_2_, O_2_^−^, and/or the hydroxyl radical (^∙^OH) [39–41]. Yet most literature on oxidative damage to human gametes is focussing on sperm and less on the oocytes or the oocyte-cumulus complex. Nevertheless, it can be assumed that this point is not less important for the fertilization process, the onset of pregnancy, and the birth of healthy offspring.

## 2. Oxidative Stress and Oocytes/Granulosa Cells

It is well-known that ROS have both beneficial and detrimental effects in terms of important regulatory functions and playing a role in the origin and progression of diseases such as cancer, neurodegenerative diseases or deleterious effects in embryo development, respectively, not only on spermatozoa [42–46], but also on all other cells in the body including cells of the female reproductive tract such as granulosa cells or oocytes [47–49].

In this context, not only are ROS levels in the follicular fluid significantly highly elevated in follicles from which poor-quality embryos derived and can therefore be regarded as a marker to determine ovarian and follicular metabolic age [50], but also the antioxidant capacity correlates positively with pregnancy after intracytoplasmic sperm injection in healthy women with endometriosis [51, 52]. Similar relationships were found in women with polycystic ovaries after IVF and ICSI [48, 53]. Even the serum and follicular fluid content of vitamins C and E may be related to the success in assisted reproduction [53].

On the other hand, in light of a proper redox balance both ROS and antioxidants transduce signals and trigger physiological events. It could be shown that ROS produced by leukocytes in the follicle at ovulation induce oocyte maturation, whereas antioxidants inhibit resumption of meiosis [54], thus suggesting a close relationship in the ovary. Thus, for healthy oocyte development a proper interplay of ROS with relevant enzymatic antioxidants such as superoxide dismutase, catalase, glutathione peroxidase, or glutathione reductase maintains the levels of reduced glutathione (GSH) and thus inhibits atresia of antral follicles and granulosa cell apoptosis [55]. After the LH surge triggering ovulation, oocyte GSH concentration increases rapidly as GSH is essential for fertilization events and early embryo development as GSH depletion prevents sperm chromatin decondensation and formation of the male pronucleus [56–58] where GSH is important for the reduction of the disulphide bridges of the protamines in the sperm nucleus [57, 59].

Apart from the direct influence in the fertilization and oocyte maturation process and embryo development outlined above, oxidative stress has been implicated in ovarian steroidogenesis and luteolysis and therefore indirectly affects female fertility [47, 60, 61].

## 3. Factors Causing Sperm DNA Damage

Factors that can cause DNA damage include apoptosis, improper DNA packaging and ligation during spermatogenesis and sperm maturation, and oxidative stress [62–66]. Reactive oxygen species (ROS) can be produced in an ejaculate by the spermatozoa via the normal leakage of electrons from the mitochondrial electron transfer chain from Complex I or III [67] or leukocytes [20, 68, 69]. Eventually, overavailability of these oxidants caused by either an overproduction of ROS or underavailability of antioxidative protection by various scavengers leads to an imbalance of the extremely sensitive redox equilibrium and thus oxidative stress [35]. The overavailability of ROS and the underavailability of antioxidants, respectively, can be caused by diseases such as genital tract infections, by unhealthy lifestyle, or by the laboratory handling of the gametes in course of an assisted reproduction treatment.

Most of the focus in the literature is on the detrimental effects of ROS on spermatozoa. A target of direct ROS action is the polyunsaturated fatty acids in the plasma membrane [70, 71] leading to lipid peroxidation and direct loss of motility. In addition, ROS can also directly damage the gametes' DNA. On the other hand, indirect action via end products of lipid peroxidation leads to the formation of carbonyl-containing compounds such as malondialdehyde, various 4-hydroxy-2-alkenals such as 4-hydroxynonenal and 2-alkenals [72] which are genotoxic and cancerogenic [73, 74], thus affecting male fertility and thereby possibly contributing to higher rates of malformations. Alternatively, ROS have the ability not only to decrease the activity of antioxidative enzymes [75], but also to damage mitochondrial DNA (mtDNA), which encodes 13 polypeptides essential for the electron transfer chain on the inner mitochondrial membrane and is, therefore, intimately involved in oxidative phosphorylation and ATP production in the mitochondria. Hence, mtDNA defects will inevitably result in a decreased mitochondrial membrane potential (ΔΨ*m*) thereby throwing the redox balance into the direction of oxidative stress. Defective mitochondrial function is essential for sperm motility [76] and has been suggested as a sensitive sperm parameter [77].

An association between male subfertility and infertility with the presence of genital tract infection and an increased number of leukocytes exists even though other scholars have on the contrary shown that seminal plasma leukocytes have no impact on the sperm fertilizing capacity [78]. The seminal plasma contains natural antioxidant such as vitamins C and E, superoxide dismutase, glutathione, uric acid, and polyamine spermine which act as a free radical scavenger. Spermatozoa depend on this scavenging system provided by the seminal plasma after normal ejaculation* in vivo* [20, 68, 69, 79–81]. Besides fertility, leukocytes negatively correlate with semen quality, PMN elastase, and ROS by secreting cytokine. Leukocytes produce 1,000 times more ROS than spermatozoa [82, 83], yet there is a stronger correlation between the percentage of ROS producing spermatozoa and sperm DNA damage than those from leukocyte derived ROS production [84]. In terms of the percentages of DNA damage, it was recommended that testicular sperm rather than ejaculated spermatozoa be used for ICSI [85].

Other factors that contribute to DNA damage are sperm storage and sperm separation techniques. Fresh semen samples were shown to have reduced levels of DNA fragmentation while the levels of DNA fragmentation increased after storage such that wet-ice freezing and snap-freezing had similar effect of the damage in comparison to cryopreservation by TEST-yolk buffer with glycerol (TYBG) [85]. From a separation technique point of view, density-gradient centrifugation, swim-up, and density-gradient centrifugation followed by a swim-up reduced the rate of DNA fragmentation compared to the increasing effect in fresh and washed semen samples, with density-gradient centrifugation step followed by a swim-up having the most significant effect [85].

## 4. Sperm Separation Techniques

Sperm separation from seminal plasma is an essential step for any assisted reproduction technique. Even* in vivo*, sperm are separated from the protective seminal fluid as the male germ cells move out of this milieu and thereby gaining fertilizing ability and the inhibiting decapacitation factors such as spermine or glycodelins, which are abundant in seminal fluid, are removed from the sperm plasma membrane [86–88].

Historically, the first sperm separation technique included up to two washing procedures in order to remove seminal plasma with subsequent resuspension of male germ cells [86–88]. This was followed by the employment of more sophisticated methods using a swim-up procedure from the washed cell pellet in order to obtain sufficient amount of motile, functionally competent spermatozoa for IVF [89]. Following these first reports on human sperm separation, more sophisticated methods were developed to obtain sufficient amounts of motile, functionally competent spermatozoa for IVF.

Sperm selection for assisted reproduction should aim to minimize the risk of abnormal sperm participating in the process of fertilization with the ideal technique being able to eliminate nonviable spermatozoa, leukocytes, bacteria, and other sources of contamination [90]. According to Henkel [1] criteria for a “good” sperm selection include the following: elimination of seminal plasma, decapacitation factors and debris, ROS producing sperm, leukocytes, and bacteria, enrichment of functional sperm, cost-effectiveness, ease, and quickness to be performed, and allowance for larger volumes of ejaculates to be processed.

In each case, care must be taken when employing any of the different methods, also taking the specific situation of the individual patient into account. Currently, the standard sperm processing techniques employed in assisted reproduction programs include simple washing, swim-up, migration and sedimentation, glass wool filtration, and density-gradient centrifugation (DGC). For the latter, different kinds of gradients such as PureSperm®, Percoll® gradients that produce varying results are used. The specific advantages and disadvantages have been reviewed [84]. Other more sophisticated and more recently developed techniques are annexin V magnetic activated cell separation which is based on the externalization of phosphatidylserine [91], hyaluronic acid- (HA-) mediated sperm selection based on the presence of HA receptors [92], electrophoretic isolation [93, 94], and the zeta method, which is based on sperm membrane electric charge [95].

With respect to finding the most appropriate method to employ in order to obtain a normal functional sperm (i.e., without damage), several studies had been conducted with no consensus reached as to which method between density-gradient centrifugation and swim-up method can be recommended [96–99]. The use of apoptotic or DNA-damaged sperm during assisted reproductive techniques (ARTs) has been linked to be one reason for suboptimal fertilization results. Particularly in cases with extremely poor semen quality, when intracytoplasmic sperm injection (ICSI) needs to be performed to achieve fertilization, the demand for the sperm separation technique lies in selecting not only the most motile spermatozoa, but also the most competent ones. The problem is that functional competence of individual sperm cells including the quality of chromatin condensation and DNA integrity cannot be assessed using normal light microscopy without using individual spermatozoa for the test. Therefore, scientists tried to find physiologic associations between visible features and the DNA quality and relate these associations to sperm separation.

Ricci et al. [100] aimed to compare the effects of density-gradient centrifugation and swim-up methods on sperm quality by the use of multiparameter flow cytometry. Their findings indicate that DGC significantly increased the mean recovery rate of viable sperm (“quantity”), while swim-up significantly lowered the mean percentage of apoptotic and necrotic sperm (“quality”). Furthermore, these authors also suggested that both preparatory methods led to obtaining a sperm population with a low percentage of apoptotic sperm. Considering the unavailability of flow cytometry in most ART laboratories, the choice of method of sperm preparation will depend on whether the sperm will be used for IUI or IVF/ICSI techniques [100].

Another study showed that fresh and washed semen samples had the highest levels of sperm DNA fragmentation compared to swim-up, DGC, and DGC followed by a swim-up, with the latter having the most significant effect [85]. On the other hand, other authors demonstrated that elimination of apoptotic-like spermatozoa from semen is not very effective after DGC [101–103]. DGC and the swim-up technique have different efficiencies in removing single- or double-stranded DNA breaks [104], of which the former is quite effective in isolating spermatozoa with singular characteristics such as large size telomere [105].

In a different study, DGC brought about a significant reduction in the baseline level of sperm DNA fragmentation but is deleterious to the sperm DNA longevity after freezing and thawing in comparison to neat semen samples [106]. Hence, these authors proposed a direct sperm wash using a standard semen extender and direct sperm isolation using polyvinyl pyrrolidone (PVP), thereby avoiding centrifugation. This principle was first described by Shekarriz et al. [107] who showed that centrifugation time is an essential factor that causes sublethal damage to spermatozoa due to oxidative stress.

Translocation of phosphatidylserine (PS) from the inner to the outer leaflet of the plasma membrane takes place during early stages of apoptosis [108]. Considering that annexin V has a high affinity for PS and binds to externalized PS, this test serves as an early marker of apoptosis [109]. According to Hoogendijk et al. [102] the use of fluorescing conjugate annexin V and flow cytometry with a strict sperm morphology assessment is a noninvasive method that can be effectively used to separate annexin V-negative and V-positive sperm subpopulations. These findings also suggested that annexin V-negative spermatozoa have a morphologically superior quality compared to the annexin V-positive subpopulation [102].

Bungum et al. [110] recommended that sperm DNA analysis by means of the SCSA should be performed on raw semen aliquots, as elevated DFI identified in neat semen may reflect chromatin abnormalities within an entire sperm population and are not completely eliminated by density-gradient centrifugation or swim-up [111]. Hence, the DFI in neat semen is regarded as predictive of the treatment outcome of ART whereas the determination of this parameter in DGC samples would not be predictive of pregnancy outcome [18, 110–112].

Similar to Jackson et al. [85], Toro and coworkers [113] showed a significant increase in DFI in samples incubated for 2–4 hours at room temperature in comparison to nonincubated (fresh) semen samples. The implication of this finding for ART is that the DFI can be higher in processed semen samples, that is, incubated at room temperature or cryopreserved. In addition, in a fixed aliquot used for DNA fragmentation testing before insemination the DNA damage as determined by means of the SCSA can be different from that in the sample directly used for oocyte insemination [113]. Hence, unnecessary incubation of semen in the laboratory should be avoided, as increased sperm DNA fragmentation was observed during aerobic incubation of semen and after semen cryopreservation [113].

Since the 1960s, cryopreservation of human spermatozoa has been a routine practice in many assisted reproductive technology laboratories [114] and has become an integral part of ARTs [115]. However, this technique poses the risk of oxidative stress with increased apoptotic DNA fragmentation regardless of the sperm concentration, yet the percentage of DNA-damaged sperm is higher in oligozoospermic men [116]. It appears that particularly the thawing process of cryopreserved sperm results in increased oxygen radical induced damage that leads to sperm DNA fragmentation [113]. These effects of ROS on DNA integrity include abasic sites, cross-linking, modification of nitrogenous bases, and DNA strand breakages [117, 118].

Repeated freezing and thawing cycles are usually offered to patients in order to maximize the use of the available sperm for reasons that may include maximizing the usage of samples obtained for cryopreservation before the treatment of cancer or other diseases, from patients with severe oligozoospermia or intermittent azoospermia, sexual dysfunction, or for the purpose of cost-effectiveness [119]. Several studies have demonstrated the detrimental effects of repeated cycles of freezing and thawing to not only decrease the percentage of motile and viable sperm but also increase the percentage of sperm with DNA damage [120–122]. On the other hand, repeated freezing and thawing up to three cycles produced similar level of risk with respect to sperm DNA damage in comparison to a single cycle, provided the samples are refrozen in their original cryoprotectant and not washed or have undergone any further treatment and are separated by DGC or swim-up before use in ART [119].

Most of the studies employed to test for DNA integrity are testing either potential (e.g., SCSA) or real (e.g., TUNEL assay) DNA fragmentation, with the relevant advantages or disadvantages [4]. Even though in the absence of fragmentation, significant DNA damage could still be detected in some genome regions [123]. In order to use a molecularly healthy semen sample for insemination, Valcarce et al. [124] recommended a quantitative PCR- (qPCR-) base technique that can be used for DNA evaluation in specific genes (PRM1, BIK, FSHB, PEG1/MEST, ADD1, ARNT, UBE3A, and SNORD116/PWSAS) that could assist in selecting and improving cryopreservation protocols used in clinics [124]. In order to investigate and compare commercially available cryoprotectant media in terms of DNA integrity of spermatozoa recovered after cryopreservation and separation using DGC, Thomson et al. [122] found no significant difference between each type of cryoprotectant to preserve DNA integrity or in its ability to predict the percentage of fragmented DNA after cryopreservation, thawing, and DGC.

Finally, considering that the current most frequently used sperm separation methods swim-up and density-gradient centrifugation are essentially limited in their selection of the most functionally competent spermatozoa, particularly in cases of poor and very poor semen quality, new procedures that could safely and efficiently select motile sperm are desirable [125] and are either in practical use such as motile sperm organelle morphological examination (MSOME) which, in combination with ICSI (IMSI) or after HA-binding (PICSI), showed ability to select functionally competent spermatozoa or in an experimental stage such as Raman spectrometry [126] or polarization microscopy [127] (for review see [5]). In the context of applying physiological criteria for the sperm selection, chemotaxis, thermotaxis, and probable oviductal contraction are also thought to be some of the physiologic mechanisms that successfully guide the sperm along the female genital tract [128]. A microchannel-based device that mimics the mammalian female reproductive tract and allows for both motility screening and chemotaxis testing simultaneously resulted in the selection of competent spermatozoa which could possibly be used for IVF to improve fertilization and pregnancy rates [125].

## 5. Effects of Lifestyle

The reproductive system has evolved over millions of years. Yet the human is unique as we are severely and rapidly not only changing our own environment, but also changing our behaviour and habits in a negative way. In addition, human spermatogenesis appears to be genetically impaired as compared to other animal species [129] and a number of mutations in fertility genes considered important in other species are evident [130, 131] and apparently make the human species essentially subfertile and more susceptible to negative environmental influences [129].

Among the lifestyle factors negatively influencing are cigarette smoking, drugs, alcohol abuse, heat exposure, or obesity. The common feature of the exposure of a man's body to these factors is the significant increase of reactive oxygen species causing oxidative stress leading to infertility as well as having significant effect on the offspring. Significant associations exist between paternal smoking and increased sperm DNA damage and elevated levels of 8-hydroxy-2′-deoxyguanosine (8-OHdG) [132, 133] caused by the high cadmium content of cigarette smoke which is known to trigger and promote DNA damage [133–135]. The DNA damage is further exacerbated by the presence of Ser326Cys polymorphism in the 8-oxoguanine DNA glycosylase 1 (OGG1) gene [136]. OGG1 is downregulated by cadmium [137]. In addition, paternal, but not maternal, cigarette smoking is positively associated with an increased risk of early childhood cancer in the progeny [9, 138, 139]. Moreover, the risk of oxidative damage to sperm in smokers is even higher as this habit causes a 48% increase in seminal leukocyte concentration and ROS [140] as well as a decrease in antioxidant levels [132, 141].

Alcohol has also been described as a potent systemic stimulator of ROS [142, 143], which would then contribute to seminal oxidative stress. These studies also reveal that alcoholic men often suffer from a lack of antioxidative defence due to insufficient diets, a fact which in turn worsens the situation. Maneesh et al. [144] reported that alcoholic men had significantly reduced plasma testosterone concentrations confirming the disturbances in the hypothalamus-pituitary-gonadal axis [145]. It also seems that chronic alcohol abuse leads to a higher risk of XY chromosome aneuploidy as compared to nondrinkers [146].

Another parameter that is significantly changed as a result of our modern lifestyle is testicular temperature. The rationale behind this thermosensitivity is that in most mammalian species, including man, the testicles are located extracorporally in the scrotum resulting in scrotal temperatures of about 34°C-35°C. In addition, it is notable that the scrotal skin has very little hairs and numerous sweat glands, which ensure that the evaporating moist is cooling down the scrotum including the testicles. Moreover, the pampiniform plexus represents an effective heat exchanger by means of a counter-flow mechanism which cools down the arterial blood inflow into the testes (for review see [147, 148]). These mechanisms, together with the actions of the dartos and cremaster muscles, lead to effective testicular thermoregulation (for review see [149]). It is thought that lower scrotal temperatures reduce oxidative damage to the sperm DNA and lower the metabolic rate in the epididymis leading to less mutations and therefore to less oxidative stress, respectively [147, 150, 151]. Numerous studies revealed that both Sertoli cells and the process of spermatogenesis* per se* are sensitive to elevated temperatures, particularly the steps of the transition from gonocytes to spermatogonia A_dark_, as well as from primary to secondary spermatocytes [152–154].

The fact that many people have jobs with sedentary positions such as office workers and taxi or long-distance drivers or many men are occupationally exposed to high temperatures, for example, in the welding or metal manufacturing industry or in bakeries, and, also, regular wet heat exposure of the testicles in Jacuzzis, saunas, or hot baths can have a significant negative effect on semen quality [155]. The latter exposure appears to be reversible as studies of Jung et al. [156, 157] have shown that nocturnal scrotal cooling can improve semen quality. Well-known pathologies which lead to elevated testicular temperatures are cryptorchidism and varicocele of which both conditions have been shown to be a cause of sperm DNA fragmentation due to induction of apoptosis with subsequent consequences for the developing embryo [158, 159].

Last but not least, overweight, obesity, and metabolic syndrome not only are increasing problems worldwide contributing to the overall burden of other chronic illnesses and causing major conditions such as cardiovascular diseases, but also significantly affect male fertility via various possible mechanisms. Particularly, obesity and the metabolic syndrome are considered to cause a systemic inflammatory condition with increased levels of C-reactive protein and inflammatory cytokines [160] and ROS [161]. Several authors [162–165] could demonstrate the significant negative effect of obesity on sperm count, motility, and function. Significant differences between metabolic syndrome patients and normal fertile donors were found not only for sperm motility or DNA fragmentation, but also for serum and seminal inflammatory cytokines such as TNF*α*, IL-1*β*, IL-6, and IL-8 with values being significantly higher in seminal plasma (Leisegang et al., unpublished). Thus, it appears that these inflammatory cytokines* per se* stimulate lipid peroxidation [166, 167], a process which is triggered and propagated by ROS [168], and in turn cause DNA damage. On the other hand, according to a recent small study including six obese men by Faure et al. [169], it appears that obesity is a correctable lifestyle factor as significant loss of abdominal fat through a lifestyle program led to significantly decreased sperm DNA fragmentation, increased serum testosterone levels, decreased seminal oxidative stress by increased superoxide dismutase levels, and, most importantly, pregnancy in all spouses included in the study.

## 6. Conclusion

Since human lifestyle and behaviour and environmental pollution significantly affect male reproductive functions including the fertilizing ability of spermatozoa, more and more couples are suffering from male infertility posing an increasing global problem. As a result, assisted reproductive techniques had been developed in which scientists and clinicians try to select the most competent spermatozoa to be used for the fertilization process. However, all these efforts are only dealing with the symptoms and consequences of the problem as many of the factors causing oxidative stress to the male germ cells are modifiable either by avoidance of exposure to environmental toxicants or by behavioural changes (e.g., stopping smoking and drinking, wearing loose underpants) or by loss of weight and following a healthy diet [170]. Alternatively, oxidative stress can be reduced by taking clinically formulated antioxidant supplements which if correctly administered to the patient can improve the success rate of reproduction. Yet uncontrolled intake of the so-called healthy supplements can also cause harm and significant adverse effects [35, 44, 171]. Therefore, a complete evaluation, not only of the individual patient, but also of the sperm nucleus quality, should be mandatory [172].
